# Viral aetiology influenza like illnesses in Santa Cruz, Bolivia (2010–2012)

**DOI:** 10.1186/1743-422X-11-35

**Published:** 2014-02-24

**Authors:** Julie Delangue, Yelin Roca Sanchez, Géraldine Piorkowski, Maël Bessaud, Cécile Baronti, Laurence Thirion-Perrier, Roxana Loayza Mafayle, Cinthia Avila Ardaya, Gabriela Añez Aguilera, Jimmy Revollo Guzman, Javier Lora Riera, Xavier de Lamballerie

**Affiliations:** 1 IRD French Institute of Research for Development, EHESP French School of Public Health, UMR_D 190 “Emergence des Pathologies Virales”, Aix Marseille Univ, 13005 Marseille, France; 2 Inmunologia y Biologia molecular, Centro National de Enfermedades Tropicales, Av 26 de Febrero esquina Centenario 2do Anillo, Santa Cruz, Bolivia

**Keywords:** Epidemiology, Influenza like illness, Bolivia, South America, Influenza A(H1N1)pdm2009, Influenza phylogeny, Respiratory infections

## Abstract

**Background:**

Acute respiratory infections represent a serious public health issue worldwide but virological aetiologies of Influenza Like Illnesses (ILIs) remain largely unknown in developing countries. This study represents the first attempt to characterise viral aetiologies of ILIs in Bolivia.

**Methods:**

It was performed in Santa Cruz city from January 2010 to September 2012, based on 564 naso-pharyngeal swabs collected in a National Reference Laboratory and real-time PCR techniques, viral cultures and phylogenetic analyses.

**Results:**

50.2% of samples were positive for at least one virus with influenza viruses (Flu A: ~15%; Flu B: ~9%), rhinoviruses (~8%), coronaviruses (~5%) and hRSV (~4%) being the most frequently identified. The pattern of viral infections varied according to age groups. The elucidation rate was the highest (>60%) amongst patients under 10 yo and the lowest (<40%) amongst patients ≥60 yo. Nearly 3% of samples showed dual viral infections. Epidemiological peaks were associated with a predominant virus but generally included 30-50% of infections by different viruses. Unexpectedly, the frequency of influenza in the 0–4 yo population was very low and a complete hRSV eclipse occurred in 2011. Genetic analyses indicated that distinct evolutionary lineages of Flu A(H1N1)pdm2009, Flu A/H3N2 and Flu B have co-circulated in Bolivia in the study period, originating from Central and North America, Europe, Asia and Australia.

**Conclusion:**

Our results emphasise the requirement for a reinforced epidemiological and genetic follow-up of influenza and other ILIs in Bolivia to further inform the preparation of vaccines used in the region, guide vaccination campaigns and improve the medical management of patients.

## Background

Acute respiratory tract infections (ARIs) represent a leading cause of human acute illnesses worldwide and an important contributing factor of childhood morbidity and mortality, especially under the age of 5 [1-3]. Besides the issue of bacterial pneumonia, which represents a frequent cause of mortality in paediatric populations of developing countries, there is an increasing interest in the epidemiology and characterization of Influenza Like Illnesses (ILIs), which are predominantly of viral aetiology [4]. First, ILIs are responsible for a large number of cases in all age groups (with a specifically high frequency in children) and despite their frequent association with a mild clinical presentation, their medical and socio-economical impact is enormous [5]. Second, a proportion of ILIs is associated with severe clinical evolution [6]. In adults, viruses are the putative causative agents in a third of cases of community-acquired pneumonia, in particular influenza viruses, rhinoviruses, and coronaviruses. Dual viral infections are common, and a large proportion of children have evidence of viral-bacterial co-infection [4]. Deciphering the epidemiological and pathophysiological links between viral primary infection and secondary bacterial super-infection and pneumonia currently represents a major challenge for improving the prevention and medical management of pneumonia.

Despite their importance in terms of morbidity as well as infant mortality, microbiological aetiologies of ARIs represent a complex, which has yet to be fully characterized in developed countries and remains largely unknown in developing countries. Despite recent improvements due to prospective studies launched during or following the influenza A(H1N1)pdm09 in Peru and Ecuador [7,8], the information about ILIs in the tropical Hispanic regions of South America is scarce and the epidemiology remains insufficiently characterised.

Here, we have performed the first study of ILIs in Bolivia, which covers 3 years from January 2010 to September 2012. The viral aetiology of infections was investigated based on naso-pharyngeal swabs collected in a National Reference Laboratory in the city of Santa Cruz de la Sierra and real time PCR molecular biology techniques, viral cultures and phylogenetic analyses.

## Results

### Characteristics of the sample studied

The number of samples that could be analysed each year at Centro Nacional de Enfermedades Tropicales, CENETROP was determined according to logistical considerations (257 in 2010, 163 in 2011 and 144 in 2012; total = 564). We did not attempt to build a representative sample of specimens received at CENETROP (supposedly poorly representative of ILIs in the general population of Santa Cruz), but rather examined a similar number of samples in each age group to allow a comprehensive aetiological analysis in all age classes. Our final sample has equivalent numbers of swabs in the 0–9, 10–19, 20–29 and 30–39 age classes (90 to 98 per class) but slightly lower numbers in the older classes (40–49: 70; 50–59: 59; ≥60: 61) due to the limited number of samples received at CENETROP for these age groups. As expected, it under-represents the 0–9 years old (yo) age group (which includes ~35% of eligible samples received), and over-represents the 30–39, 40–49, 50–59 and ≥60 yo age groups (Additional file 1). The general characteristics and distribution of cases are presented in Table 1. The gender ratio (M/F) is 0.84 and the history of influenza vaccination clearly shows that a limited proportion of the population received influenza vaccines, in particular in the elderly (0.53% have received at least one dose of influenza vaccine in their life in the ≥60 yo age group).

**Table 1:** Characteristics of the population studied and viral aetiologies of ILIs (January 2010 September 2012)

		**N**	**%**	**+/-CI**
Total samples		564	100.00	
Male		257	45.57	
Female		307	54.43	
Year				
	2010	257	45.57	
	2011	163	28.90	
	2012	144	25.53	
Age groups				
0-4		69	12.23	
5-9		29	5.14	
10-19		91	16.13	
20-29		95	16.84	
30-39		90	15.96	
40-49		70	12.41	
50-59		59	10.46	
≥ 60		61	10.82	
**Influenza vaccination history**		**65**	**11.52**	**2. 80**
Before 2010		5	0.89	0.78
After 2010		60	10.64	2.69
Age groups				
0-4		5	0.89	0.78
5-9		0	0.00	0.00
10-19		6	1.06	0.85
20-29		14	2.48	1.30
30-39		17	3.01	1.43
40-49		9	1.60	1.04
50-59		11	1.95	1.15
≥ 60		3	0.53	0.60
**Virus detected**		**283**	**50.18**	**5.85**
Influenza A/H1N1p		48	8.51	2.41
Influenza A/H3N2		37	6.56	2.11
Influenza B		50	8.87	2.46
Rhinovirus		44	7.80	2.31
Coronavirus E229 & OC43		28	4.96	1.84
RSV		23	4.08	1.67
Adenovirus		18	3.19	1.47
Metapneumovirus		12	2.13	1.20
Bocavirus		9	1.60	1.04
Parainfluenzavirus 1&3		9	1.60	1.04
Enterovirus and Parechovirus		5	0.89	0.78
**Total dual viral infection**		**16**	**2.84**	**1.39**
Adenovirus - Influenza A		3	0.53	0.60
Adenovirus - Coronavirus E229 & OC43		2	0.35	0.49
Bocavirus - Rhinovirus		2	0.35	0.49
Coronavirus E229 & OC43-Rhinovirus		2	0.35	0.49
Influenza A - Bocavirus		2	0.35	0.49
Influenza A - Adenovirus		1	0.18	0.35
Influenza A - Coronavirus E229 & OC43		1	0.18	0.35
Metapneumovirus - Rhinovirus		1	0.18	0.35
Enterovirus and Parechovirus - Adenovirus		1	0.18	0.35
Enterovirus and Parechovirus - Influenza A		1	0.18	0.35

### Virological results in age groups

**Figure 1:**
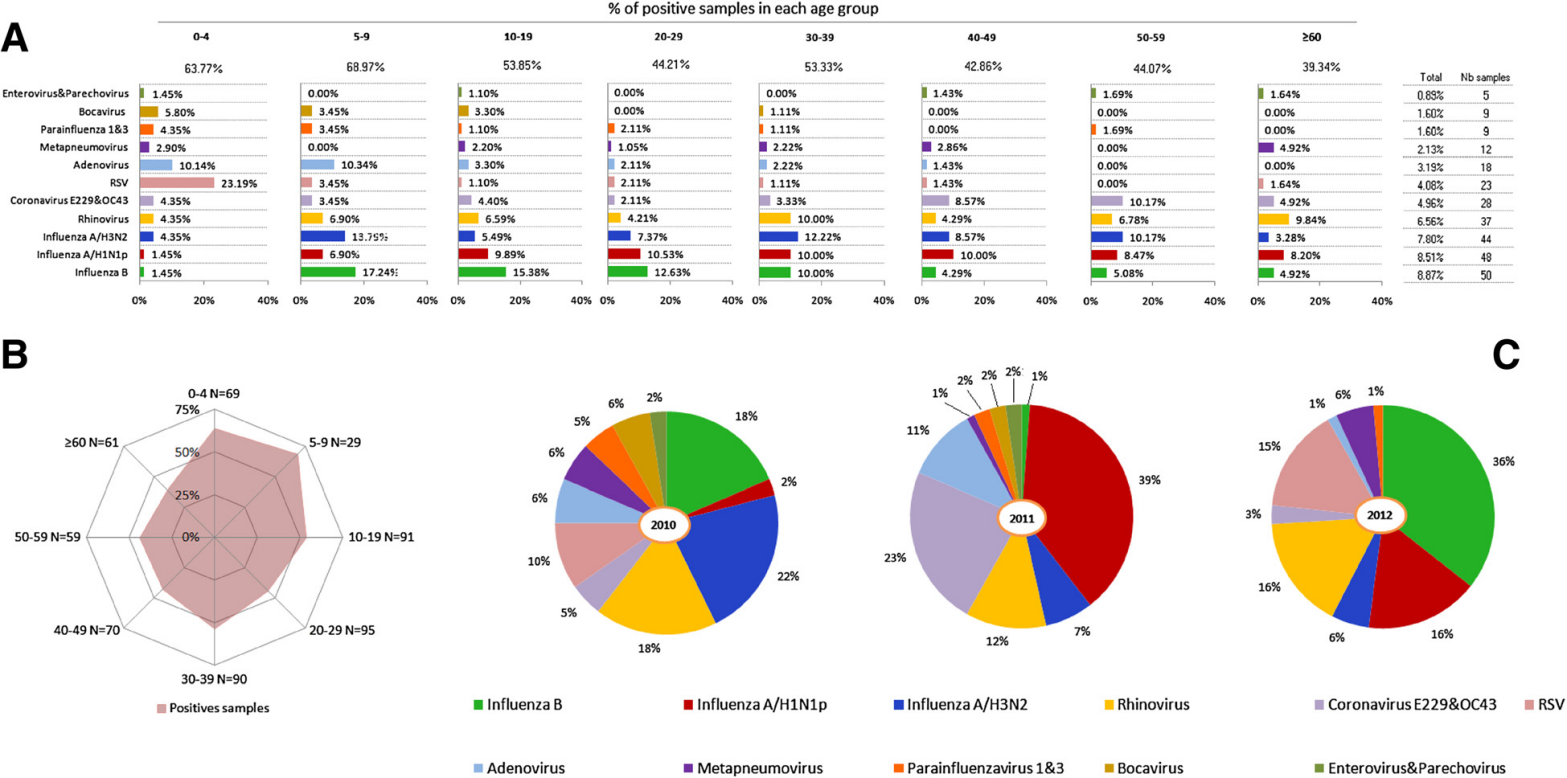
**Global distribution of viral aetiologies according to age group and year of collection. A**- Viral aetiologies in age groups. **B**- Distribution of positive and negative samples in age groups. **C**– Yearly assessment of viral diagnoses.

Of the 564 samples tested, 283 (50.2%) were positive for at least one virus (Table 1) with influenza viruses (Flu A: ~15%; Flu B: ~9%), rhinoviruses (hRV) (~8%), coronaviruses E229&OC43 (hCoV E229&OC43) (~5%) and respiratory syncytial virus hRSV (~4%) being the most frequently identified. The pattern of viral infections varied according to age groups (Figure 1A). The elucidation rate was the highest (>60%) in samples from patients under the age of 10 yo and the lowest (<40%) in patients ≥60 yo (Figures 1A and B). Since all samples originated from symptomatic patients, this may be due either to the higher prevalence of the viruses tested or to the higher level of viral shedding in children.

The 0–4 yo age group displayed specific epidemiological characteristics with the majority of hRSV infections (infection rate: 23.19%) and a remarkably low proportion of influenza infections: <7.5% compared with ~38% in the 5–9 yo age group, ~26.5% in the 10–59 yo age classes and ~16% in those ≥60 yo.

Adenoviruses were most frequently identified in children under the age of 10, bocaviruses in patients under the age of 20 and hCoV E229 & OC43, although present in all age groups, were more prevalent in patients ≥40 yo. Interestingly, metapneumoviruses (hMPV) were detected in both children and adults with the most elevated rate of infection in the ≥60 yo age group (~5%) and enteroviruses/parechoviruses were also found in both children and adults. Rhinoviruses were identified in all age groups (up to ~10% in patient over 60 years of age).

Nearly 3% of samples showed dual viral infections (Table 1): 8 (9.4%) of the 85 influenza A viruses, 5 (11.4%) of the 44 rhinoviruses and 5 (17.8%) of the 28 hCoV E229&OC43 infections detected were diagnosed in a situation of co-infection. Co-infections were specifically frequent in the case of adenoviruses (38.9%). Despite a high number of influenza B cases (50) no co-infection was noted. The rate of dual infections was high for bocaviruses and enteroviruses/parechoviruses (≥40%) and low for metapneumoviruses and parainfluenzaviruses (<10%) but this was based on a limited number of cases. The median age was not different in patients with single or dual infections.

### Temporal distribution of virological results

**Figure 2:**
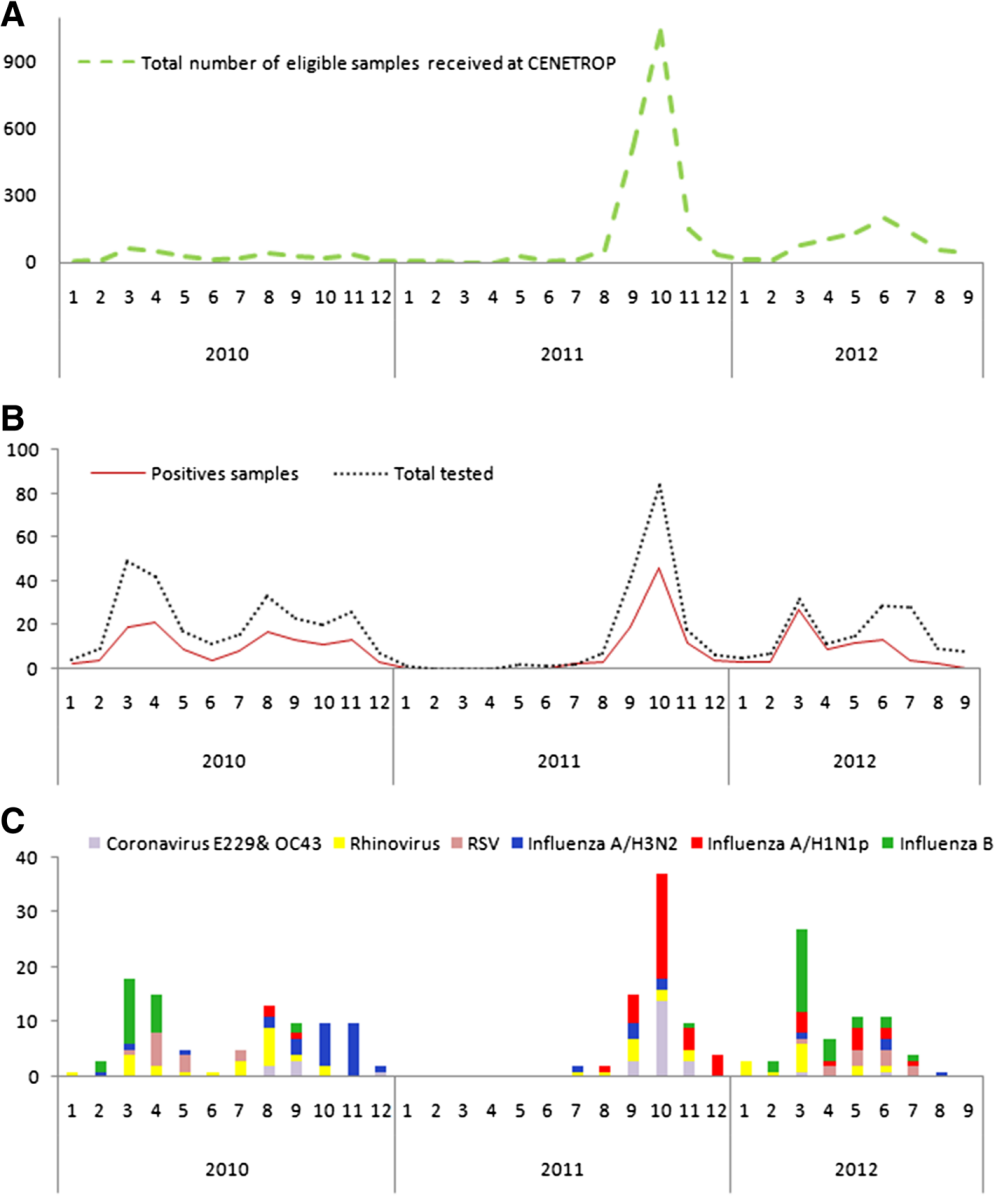
**Temporal distribution of samples. A**- The dash green curve represents nasal swabs received at CENETROP. **B**- The black dotted line represents samples tested, and the red curve represents positives (at least one virus detected). **C**– Weekly distribution of positives for Flu A/H3N2, Flu A(H1N1)pdm09, Flu B, rhinoviruses and coronaviruses E229 & OC43.

The curve of positive samples followed that of samples tested (Figure 2B) and that of samples received at CENETROP (Figure 2A). Peaks of detection matched peaks of reception of samples, which globally followed the seasonal subtropical pattern of ILIs observed in Bolivia, with one peak during the austral autumn and a second during the austral winter season. In 2010, a great number of samples could be studied (257) allowing to precisely follow several peaks of activity corresponding to a mosaic of viruses including Flu B, hRSV, hRV, Flu A A/H3N2 and A(H1N1)pdm09 and hCoV E229&OC43. In 2011 and 2012, the number of samples studied annually was lower (163 and 144, respectively), but still sufficient to elucidate the main peaks of activity with a major incidence of Flu A(H1N1)pdm09 in 2011 and Flu B in 2012, co-circulating with a variety of other viruses (see Figure 2C). Epidemiological peaks were generally associated with a predominant virus (Flu B in March 2010, hRV in August 2010, Flu A/H3N2 in November 2010, Flu A(H1N1)pdm09 in October 2011 and Flu B in March 2012), but the co-circulation of other viruses was the general rule (with the exception of Flu A/H3N2 in November 2010) and peaks also included 30-50% of infections by different viruses.

This is summarised in Figure 1C that shows a clear epidemiological evolution. In 2010, following the 2009 Flu A(H1N1)pdm09 pandemic wave, influenza A infections were for the most part due to Flu A/H3N2 and associated to a large number of Flu B and rhinovirus infections and a variety of less prevalent viruses. By contrast, year 2011 was characterised by the massive come back of Flu A(H1N1)pdm09, the low number of Flu B, the emergence of a large number of hCoV E229&OC43 infections and the absence of hRSV infections. In 2012, the situation changed again with a large number of Flu B and hRSV infections and the persistence of a significant proportion of rhinovirus and Flu A(H1N1)pdm09 infections. Altogether, this provides a striking picture of a moving infection pattern, which is presumably modelled by the herd immunity in the most epidemiologically susceptible populations. The complete disappearance of hRSV infections in 2011 represents a specific case that was never encountered by the authors in Europe.

### Phylogenetic analyses of influenza virus A and B sequences

Cell culture isolates of influenza A/H3N2 (N = 4), influenza A(H1N1)pdm09 (N = 19) and influenza B2 (N = 11) viruses were obtained. Eight-segment genomic sequences were produced for Flu A/H3N2 isolates (GenBank accession numbers KF612026-KF612180) and Flu A/H3N2 isolates (GenBank accession numbers KF612181-KF612211). Partial sequences (segments HA, NA, NS, M) were produced for Flu B isolates (GenBank accession numbers KF612212- KF612267). The corresponding sequences were used for further phylogenetic analyses.

#### Influenza B virus

**Figure 3:**
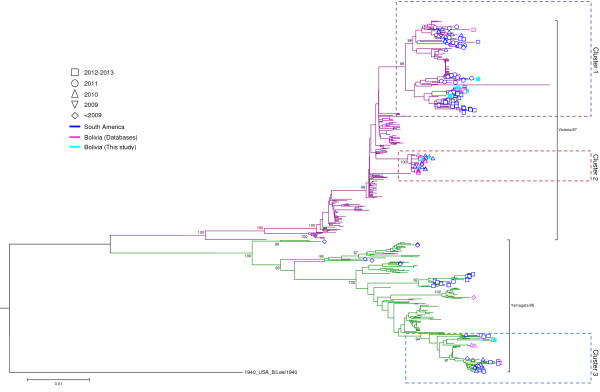
**Phylogenetic analysis of Haemagglutinin (HA) coding sequence of 485 influenza B viruses.** Main bootstrap values are indicated at forks. The Victoria/87 and Yamagata/88 lineages are indicated in pink and green, respectively. The tree includes 474 complete coding sequences from GenBank and 11 complete coding sequences from this study, GenBank accession numbers KF612026-KF612180. Clusters 1, 2 and 3 are detailed in Supplementary files (Additional file 2, Additional file 3 and Additional file 4 respectively).

Influenza B viruses are divided into two antigenic lineages (Victoria/87 and Yamagata/88) since 1983. Both of them frequently co-circulate during epidemic periods [9] and segment reassortment between strains of both lineages is frequent, making it difficult to produce robust complete-genome phylogenetic analyses. Here, evolutionary trees were reconstructed from the alignment of 485 HA sequences and showed that ten out of the eleven 2010 and 2012 Bolivian Flu B strains studied were in the Victoria/87 lineage, and one (2012) in the Yamagata/88 lineage. Victoria-related Bolivian isolates appear in two distinct clusters, together with other South American isolates (Figure 3). The first cluster, supported by a 98% bootstrap resampling value (Additional file 2), includes the 2012 Bolivian isolates, together with a vast majority of Central and South American isolates in the 2010–2012 period and a limited number of isolates from different origin (Asia, North America, Australia). According to the tree topology, the 2012 South American cluster may originate from 2010 Australian isolates (observed both at the root and within this cluster). The second cluster, supported by a 100% bootstrap value (Additional file 3), includes the 2010 Bolivian isolates, together with other Bolivian and South American isolates in the same period. The third cluster (within the Yagamata lineage), supported by a 94% bootstrap value (Additional file 4), seems to be issued from 2007 Asian isolates and includes one 2012 Bolivian isolate, together with a variety of other Bolivian, Central and South American, North American and Asian isolates in the 2007–2012 period.

Altogether, these results demonstrate the co-circulation of viruses from different lineages in Bolivia and the existence of a global epidemiological system in which influenza B of various origin (presumably most frequently from Australia, Asia and North America) circulate in Central and South America.

#### Influenza A/H3N2

**Figure 4:**
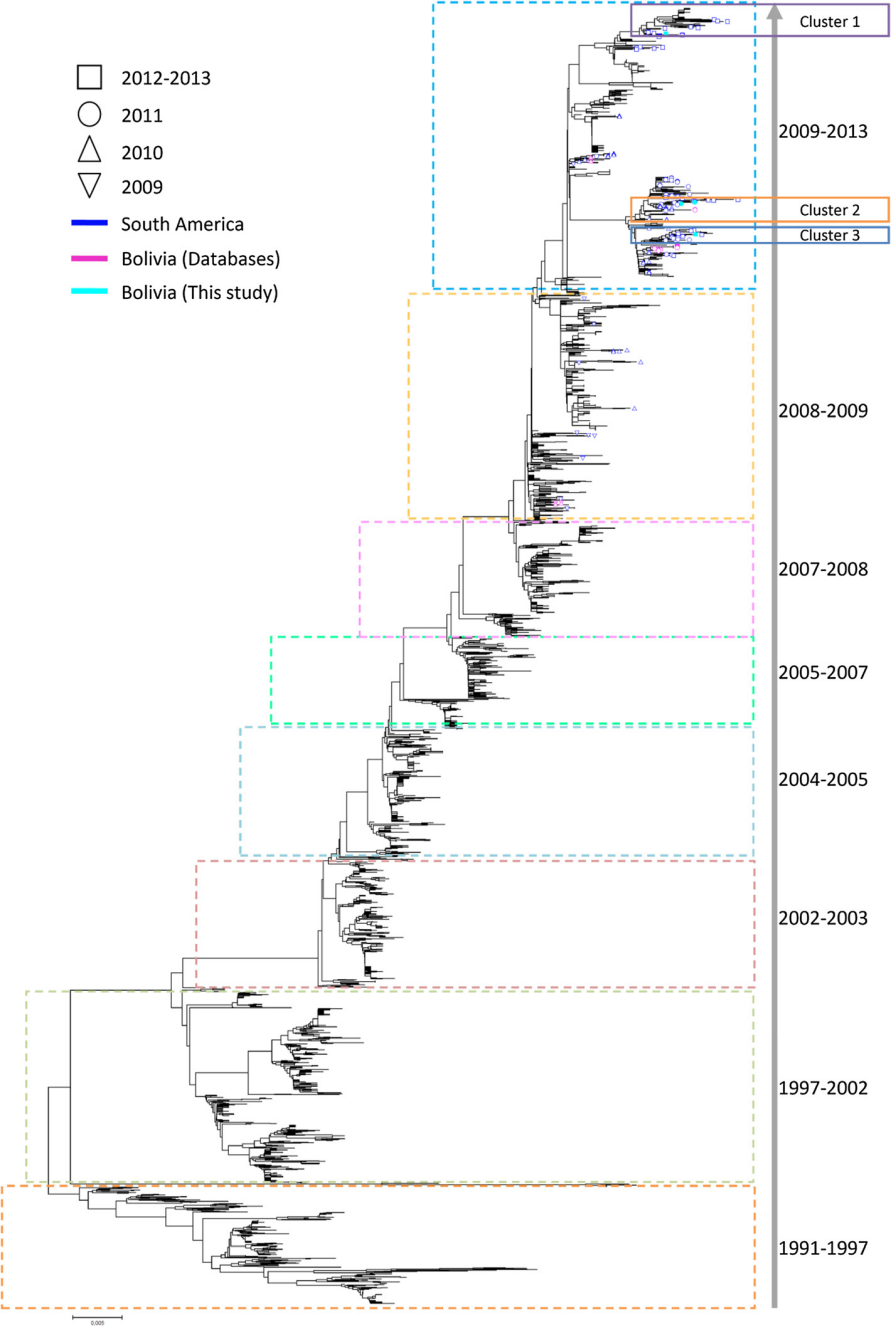
**Phylogenetic analysis of Haemagglutinin (HA) of 2,643 influenza A/H3N2 viruses.** The tree includes 2,639 complete coding sequences from GenBank and 4 complete coding sequences from this study, GenBank accession numbers KF612181-KF612211. Clusters 1, 2 and 3 are detailed in Supplementary files (Additional file 5, Additional file 6 and Additional file 7 respectively).

Figure 4 presents a tree reconstructed from 2,643 HA sequences. It illustrates the genetic drift of H3N2 viruses since 1991, but also the limited information available regarding South American strains before 2008.

Sequences from H3N2 2011–2012 Bolivian isolates appear in 3 clusters in the so-called Victoria/208 lineage. Cluster 1 (Additional file 5) includes a 2012 Bolivian isolate, together with isolates of various geographical origins. The 2012 South American isolates group with 2011 Asian and Australian isolates and may therefore be epidemiologicaly linked. Cluster 2 (Additional file 6) includes a 2011 Bolivian strain together with 2011 and 2012 South American isolates. Cluster 3 (Additional file 7) includes 2012 South American isolates related to 2011 Brazilian isolates (low bootstrap values).

These data suggest that variants belonging to distinct evolutionary clusters have circulated simultaneously in South America during the 2011 and 2012 influenza seasons. This information is relevant for optimising the formula of the seasonal influenza vaccine in order to provide efficient protection [10].

#### Influenza A(H1N1)pdm09

**Figure 5:**
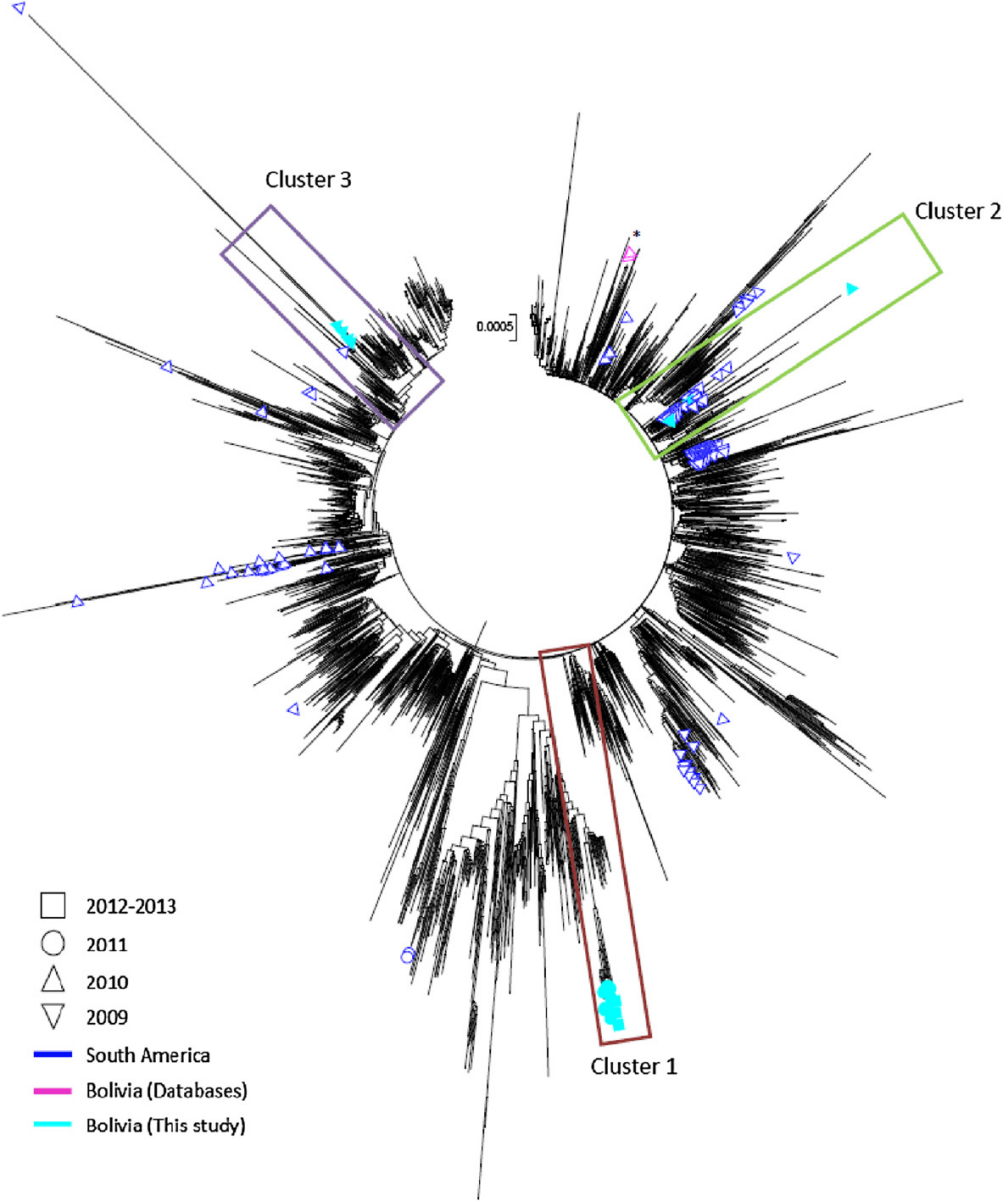
**Phylogenetic analysis of complete coding sequences of 3,412 influenza A(H1N1)pdm09 viruses.** The tree includes 3,393 complete coding sequences from GenBank and 19 complete coding sequences from this study, GenBank accession numbers KF612212- KF612267. (*): strain A/La Paz/WR0096T/2009. Clusters 1, 2 and 3 are detailed in Additional files (Additional file 8, Additional file 9 and Additional file 10 respectively).

Figure 5 presents a tree reconstructed from 3,412 complete genomic influenza A(H1N1)pdm09 sequences in the 2009–2013 period. Bolivian isolates appear in 3 distinct clusters.

Cluster 1 (Additional file 8) includes 2011 and 2012 Bolivian isolates. The 2011 Bolivian isolates were collected from October to December and are related to German strains collected in February 2011, suggesting the circulation in Bolivia during the 2011 (South hemisphere) influenza season of viruses derived from those that circulated in Europe at the end of the previous (North hemisphere) influenza season. The presence in the same cluster (100% bootstrap support) of Bolivian strains that circulated during the first 2012 influenza epidemic peak (March 2012, Figure 2) indicates that the virus has been continuously circulating at low level after November 2011 and reveals the possible persistent viral circulation over long periods (possibly a one-year period –from August 2011 to July 2012- according to Figure 2). These strains cluster with a number of Bolivian and South American strains for which only HA sequences are available (data not shown).

Clusters 2 and 3 are poorly informative (Additional file 9 and Additional file 10, low bootstrap supports). Cluster 2 includes 2009 and 2010 Bolivian isolates together with 2009 South American, Asian and North American isolates and cluster 3 includes 2009 Bolivian isolates together with 2009 South American, North American and European isolates. In addition, a complete genomic sequence available for a 2009 isolate made in La Paz, Bolivia (strain A/La Paz/WR0096T/2009) is located in a different cluster of the evolutionary tree (see Figure 5).

Altogether, the main information arising is that several evolutionary lineages co-circulated in Bolivia during the 2009 H1N1 pandemic wave.

## Discussion

This study represents the first attempt to characterise the viral aetiology of ILIs in Bolivia and the first in South America in the post-influenza A(H1N1)pdm09 period. It was not designed for providing a representative picture of the general Bolivian population: (i) samples originated from the region of Santa Cruz only (this item is further discusses below); (ii) samples sent to CENETROP were not expected to faithfully represent ILIs in the region of Santa Cruz, with obvious recruitment biases such as clinical severity or access to public medical care; (iii) sampling was performed with the objective of obtaining equivalent numbers of cases in all age classes.

The information obtained should therefore be analysed with precautions. Firstly, the great year-to-year variation of infection epidemiological patterns is a reminder that the epidemiology of ILIs should be studied over long periods of time. Each of the three years studied here would have provided, if analysed separately, a partial view of the situation and would have lead to erroneous conclusions (*e.g.*, the absence of hRSV infections in Bolivian children extrapolated from 2011 data). The analysis of a 3-year period is expected to smooth such epidemiological jolts but an extended observation period would have undoubtedly revealed more information about the viruses that circulated at low rates in the period studied (*e.g.*, parechoviruses, parainfluenzaviruses). Secondly, Bolivia includes territories with diverse geographical, climatic and ecological characteristics. Santa Cruz is located in an eastern low-altitude area with a humid tropical climate whilst La Paz is in a western Andean high-altitude area with a colder and dryer climate [11]. This may explain important epidemiological differences observed between both cities, as exemplified by the case of influenza A(H1N1)pdm09: in 2010, we observed a very limited number of Flu A(H1N1)pdm09 infections in Santa Cruz whilst the PAHO surveillance system reported an intense circulation of the virus in La Paz during the first months of the year (PAHO surveillance website: http://ais.paho.org/phip/viz/ed_flu.asp); in 2011, a large outbreak occurred in Santa Cruz (see Figures 1 and 2), whilst only a few cases were observed in La Paz; in 2012, an important outbreak was observed in La Paz, but not in Santa Cruz. This clearly indicates that epidemiological data collected in Santa Cruz cannot be extrapolated easily to the Andean regions of Bolivia. Divergent influenza epidemiological patterns according to geographical distribution were finely documented in a previous Peruvian study [7].

Despite the fact that the local epidemiology of ARIs is modelled by herd immunity and environmental factors, the global picture of ARIs that arises from our study conforms with that observed in different regions of the world. In particular, the choice of the pathogens tested seems to be relevant since (i) the rate of elucidation is close to what is observed in France, using the same molecular assays (authors’ personal data), (ii) peaks of detection match the ILI epidemiological peaks and (iii) in previous studies performed in Brazil, the different viruses identified in the current study were detected, confirming their circulation in the region [12-16]. The distribution of cases in age groups is also essentially similar with what was reported elsewhere, *e.g.*, in Ecuador [8]. The most surprising items, which deserve investigations on an extended period of time, were the unusually low frequency of influenza infections in the 0–4 yo age group and the complete RSV eclipse in 2011. However, precise comparison with data collected in South America is most often difficult, *e.g.*, with a molecular study in Ecuador [8] and a previous large study in Peru [7] (different periods investigated, viruses detected using immunofluorescence tests, parechoviruses, coronaviruses, metapneumoviruses and rhinoviruses not tested).

The genetic characterisation of influenza strains indicates that viruses belonging to distinct evolutionary lineages of Flu A(H1N1)pdm09, Flu A/H3N2 and Flu B have co-circulated in Bolivia. Interestingly, an increasing number of influenza sequences has been made available since 2008 and phylogenetic reconstructions revealed, on the one hand, the existence of South American evolutionary clusters of influenza strains, and on the other hand a complex epidemiological relationship with the rest of the world. Trees suggested the circulation in Bolivia during the study period of viruses originating from Central and North America, Europe, Asia and Australia.

## Conclusion

This study provided the first systematic and broad-spectrum information regarding the viral aetiology of ILIs in Bolivia. A continued effort is needed to accurately characterise the epidemiology of ILIs in the different regions of the country to guide vaccination campaigns for at-risk populations and improve the medical management of patients. In accordance with previous investigations performed in Peru [7], our results emphasise the requirement for a reinforced genetic follow-up of influenza strains circulating in South America to further inform the preparation of vaccines used in the region.

## Methods

### Case definition, population and samples

The recruitment of cases was operated following the broad spectrum WHO case definition of ILIs: anybody with fever of 37.8°C or higher, associated with one respiratory symptom was identified as ILI infection and eligible for investigation. We used naso-pharyngeal swabs sampled in hospitals and health centres in the region of Santa Cruz and sent to the CENETROP (Centro Nacional de Enfermedades Tropicales, Santa Cruz, Bolivia) for diagnosis from January 2010 to September 2012. All specimens had been sampled between day 0 and day 4 following clinical onset, sent to the laboratory at room temperature using a universal transport media and kept at -20°C upon reception. All patients or their legal representative gave oral consent for the shipment of samples to CENETROP and the detection of respiratory pathogens. To provide a preliminary report of viral aetiologies in all age groups (*i.e.*, information that may be translated easily for Public health purposes), a random selection of an equivalent number of samples was operated in age groups (0–9, 10–19, 20–29, 30–39, 40–49, 50–59 and ≥60 years of age) for each year of the study.

### Virus identification

Aetiological identification was performed using TaqMan probe-based real-time PCR and RT-PCR protocols. Samples were spiked by MS2 and T4 phages, used as internal controls [17]. RNA and DNA were co-extracted from 200 μL of each sample and eluted in a 90 μL final volume (QIAcube automate and QIAamp MinElute Virus Spin Kit, both from Qiagen). Random hexaprimer reverse transcription of RNA genomes was performed using the TaqMan Transcriptase reverse reagent kit (Roche). Amplifications were performed in a final volume of 25 μL, using 3 μL of nucleic acid solution, the Fast qPCR MasterMix with no Rox (Eurogentec) and a Bio-Rad CFX96 Touch™ Real-Time PCR Detection System.

Amplification protocols included the specific detection of seasonal Influenza A viruses (Flu A, [18]), pandemic A/H1N1(2009) influenza virus (Flu A(H1N1)pdm09, ( [19])), Influenza B virus (Flu B, [18]), Parainfluenzaviruses 1&3 (hPIV 1&3, [20]), Respiratory syncytial viruses A and B (hRSV A, hRSV B, [21]), Coronaviruses E229&OC43 (hCoV E229&OC43, [22]), Rhinovirus (hRV, [23], Adenoviruses (hAdV, [24]), Enteroviruses & Parechoviruses (hEVs, hPeV, [25]), human metapneumoviruses A and B (hMPV, [26]), human Bocaviruses (hBoV, [27]).

### Culture

Samples providing a strong positive real-time PCR result (Ct < 30) for influenza A or B viruses were used for virus isolation using MDCK cells and a standard isolation protocol (Additional file 11).

In addition to strains isolated in the course of the current study, 6 influenza A(H1N1)pdm09 virus strains isolated at CENETROP in the June-August 2009 period were sequenced to reinforce phylogenetic analyses (see below).

### Sequencing

200 μL of clarified supernatant from Influenza A or B positive cell cultures were extracted using the EZ1® Qiagen instrument and the EZ1® Virus Mini Kit v2.0 without RNA Carrier (Qiagen). All 8 segments of influenza A virus were amplified in a single reaction using the SuperScript® III One-Step RT-PCR System with the Platinum® Taq High Fidelity Kit and the universal primers univ12 and univ13 [28].

To obtain influenza B virus sequences, we used a similar protocol and one set of primers (BM-NS-1 and BM-NS-2) to amplify the HA, NA and NS segments [29] and another set of newly designed primers (M-2eFor: AGAAGYASAGCATTTTCTTGTGA and M-2eRev: TAAACACCCACATYCCAAACGT) to amplify the M segment.

PCR products were purified (Nucleofast PCR plate, Machrey Nagel) and sequenced using the NGS Ion Torrent technology: briefly, DNA concentrations were adjusted to 200nM (Qubit® dsDNA BR Assay Kit and Qubit® 2.0 Fluorometer) and a library of barcoded 100–200 nt fragments was built using the Diagenode Bioruptor UCD-200, the Xpress™ Barcode Adapters kit and the AB Library Builder™ (Life Technologies). Clonal amplification was performed using the Ion PGM™ Template OT2 200 Kit and the Ion OneTouch™ 2 Instrument (both from Life Technologies). Final sequencing was performed using the Personal Genome Machine® (PGM™) System, the Ion PGM™ Sequencing 200 Kit v2 and the Ion 316™ Chip Kit (Life Technologies).

### Processing of sequences and phylogenetic analyses

To produce consensus sequences for each isolated strains, reads obtained from using the Ion Torrent protocol were processed using the CLC Genomics Workbench software 6.04 (CLCbio, Aarhu, Denmark) and a home developed program and then mapped onto concatenated reference sequences.

In the case of A/H1N1(2009) phylogenies, concatenated coding sequences of the eight segments from the complete genomic sequences produced as reported above from Bolivian strains were aligned with complete genomic sequences retrieved from databases (total: 3,412 genomic sequences). In the case of A/H3N2 and influenza B phylogenies we collected complete genomic sequences from databases but used only the HA gene sequences to produce alignments (A/H3N2: 2,643 sequences; influenza B: 485 sequences). Alignments were conducted using the MUSCLE programme [30], implemented in the Influenza Research Database (Multiple Sequence Alignment: http://www.fludb.org/brc/) and subsequently used to perform phylogenetic analyses using the MEGA 5.05 programme [31], the Jukes Cantor algorithm for distance calculation and the Neighbour joining method for tree building.

## Abbreviations

CENETROP: Centro Nacionales de Enfermedades Tropicales; ARIs: Acute respiratory tract infections; ILIs: Influenza like illnesses; HA: Haemagglutinin; Flu A: Seasonal influenza A viruses; Flu A(H1N1)pdm09: Pandemic influenza A/H1N1(2009) virus; Flu B: Influenza B virus; hPIV 1&3: Parainfluenzaviruses 1&3; hRSV A hRSV B: Respiratory syncytial viruses A and B; hCoV E229&OC43: Coronaviruses E229&OC43; hRV: Rhinovirus; hAdV: Adenoviruses; hEVs&hPeV: Enteroviruses & Parechoviruses; hMPV: Human metapneumoviruses A and B; hBoV: Human bocaviruses.

## Competing interests

The authors declare that they have no competing interests.

## Authors’ contributions

JD: Participate on sampling samples molecular biology, culture and sequencing parts and draft of paper; YRS: Participate to contributions to conception and design of project. GP: have made substantial contributions to conception and design of influenza sequencing participated in the sequence treatment alignment. MB: helps analysis and interpretation of data. CB; have made substantial contributions to conception and design of influenza sequencing. LTP; have been involved in drafting the manuscript. RLM: Participate on molecular biology and viral culture. CAA: Participate on sampling samples molecular biology. GAA: Participate on sampling samples molecular biology. JRG; Participate to contributions to conception and design of project. JLR: Participate to contributions to conception and design of project. XdL: have given correction and final approval of the version to be published. All authors read and approved the final manuscript.

## Supplementary Material

Additional file 1 Distribution of the eligible and studied populations in age groups. Click here for file

Additional file 2 **Influenza B phylogeny cluster 1.** This is a magnification of Influenza B phylogeny cluster 1 (Figure 3). Legend shape represents the year of strain isolation: square for 2012; circle for 2011; triangle with the tip up for 2010, triangle with the tip down for 2009; rhombus for <2009. The colour represents the geographical origin: light blue for Bolivian strains from this study; pink for Bolivian strains from databases; dark blue for South American strains from databases. Click here for file

Additional file 3 I**nfluenza B phylogeny cluster 2.** This is a magnification of Influenza B phylogeny cluster 2 (Figure 3). Legend shape represents the year of strain isolation: square for 2012; circle for 2011; triangle with the tip up for 2010, triangle with the tip down for 2009; rhombus for <2009. The colour represents the geographical origin: light blue for Bolivian strains from this study; pink for Bolivian strains from databases; dark blue for South American strains from databases. Click here for file

Additional file 4 **Influenza B phylogeny cluster 3.** This is a magnification of Influenza B phylogeny cluster 3 (Figure 3). Legend shape represents the year of strain isolation: square for 2012; circle for 2011; triangle with the tip up for 2010, triangle with the tip down for 2009; rhombus for <2009. The colour represents the geographical origin: light blue for Bolivian strains from this study; pink for Bolivian strains from databases; dark blue for South American strains from databases. Click here for file

Additional file 5 **Influenza A/H3N2 cluster 1.** This is a magnification of Influenza A/H3N2 phylogeny cluster 1 (Figure 4). Legend shape represents the year of strain isolation: square for 2012; circle for 2011; triangle with the tip up for 2010, triangle with the tip down for 2009; rhombus for <2009. The colour represents the geographical origin: light blue for Bolivian strains from this study; pink for Bolivian strains from databases; dark blue for South American strains from databases. Click here for file

Additional file 6 **Influenza A/H3N2 cluster 2.** This is a magnification of Influenza A/H3N2 phylogeny cluster 2 (Figure 4). Legend shape represents the year of strain isolation: square for 2012; circle for 2011; triangle with the tip up for 2010, triangle with the tip down for 2009; rhombus for <2009. The colour represents the geographical origin: light blue for Bolivian strains from this study; pink for Bolivian strains from databases; dark blue for South American strains from databases. Click here for file

Additional file 7 **Influenza A/H3N2 cluster 3.** This is a magnification of Influenza A/H3N2 phylogeny cluster 3 (Figure 4). Legend shape represents the year of strain isolation: square for 2012; circle for 2011; triangle with the tip up for 2010, triangle with the tip down for 2009; rhombus for <2009. The colour represents the geographical origin: light blue for Bolivian strains from this study; pink for Bolivian strains from databases; dark blue for South American strains from databases. Click here for file

Additional file 8 **Influenza A(H1N1)pdm09 cluster 1.** This is a magnification of Influenza A(H1N1)pdm09 phylogeny cluster 1 (Figure 5). Legend shape represents the year of strain isolation: square for 2012; circle for 2011; triangle with the tip up for 2010, triangle with the tip down for 2009; rhombus for <2009. The colour represents the geographical origin: light blue for Bolivian strains from this study; pink for Bolivian strains from databases; dark blue for South American strains from databases. Click here for file

Additional file 9 **Influenza A/H1N1 cluster 2.** This is a magnification of Influenza A(H1N1)pdm09 phylogeny cluster 2 (Figure 5). Legend shape represents the year of strain isolation: square for 2012; circle for 2011; triangle with the tip up for 2010, triangle with the tip down for 2009; rhombus for <2009. The colour represents the geographical origin: light blue for Bolivian strains from this study; pink for Bolivian strains from databases; dark blue for South American strains from databases. Click here for file

Additional file 10 **Influenza A/H1N1 phylogeny cluster 3.** This is a magnification of Influenza A(H1N1)pdm09 phylogeny cluster 3 (Figure 5). Legend shape represents the year of strain isolation: square for 2012; circle for 2011; triangle with the tip up for 2010, triangle with the tip down for 2009; rhombus for <2009. The colour represents the geographical origin: light blue for Bolivian strains from this study; pink for Bolivian strains from databases; dark blue for South American strains from databases. Click here for file

Additional file 11 Sequenced sample information. Click here for file
