# Supplementary figures and images for: Viral aetiology influenza like illnesses in Santa Cruz, Bolivia (2010–2012)
Source: Virol J. 2014 Feb 24;11:35. doi: 10.1186/1743-422X-11-35 (PMC4015617; doi:10.1186/1743-422X-11-35)

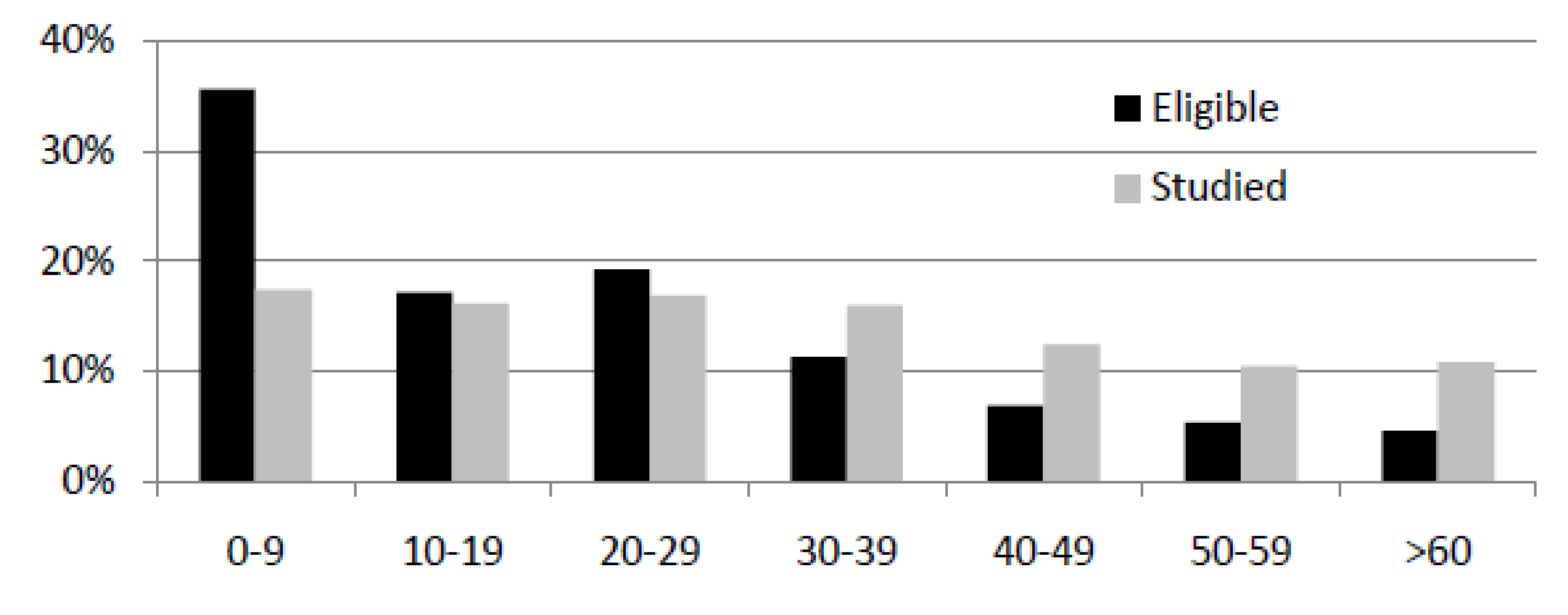

Supplement: Additional file 1 — Distribution of the eligible and studied populations in age groups. [file 1743-422X-11-35-S1.tiff]

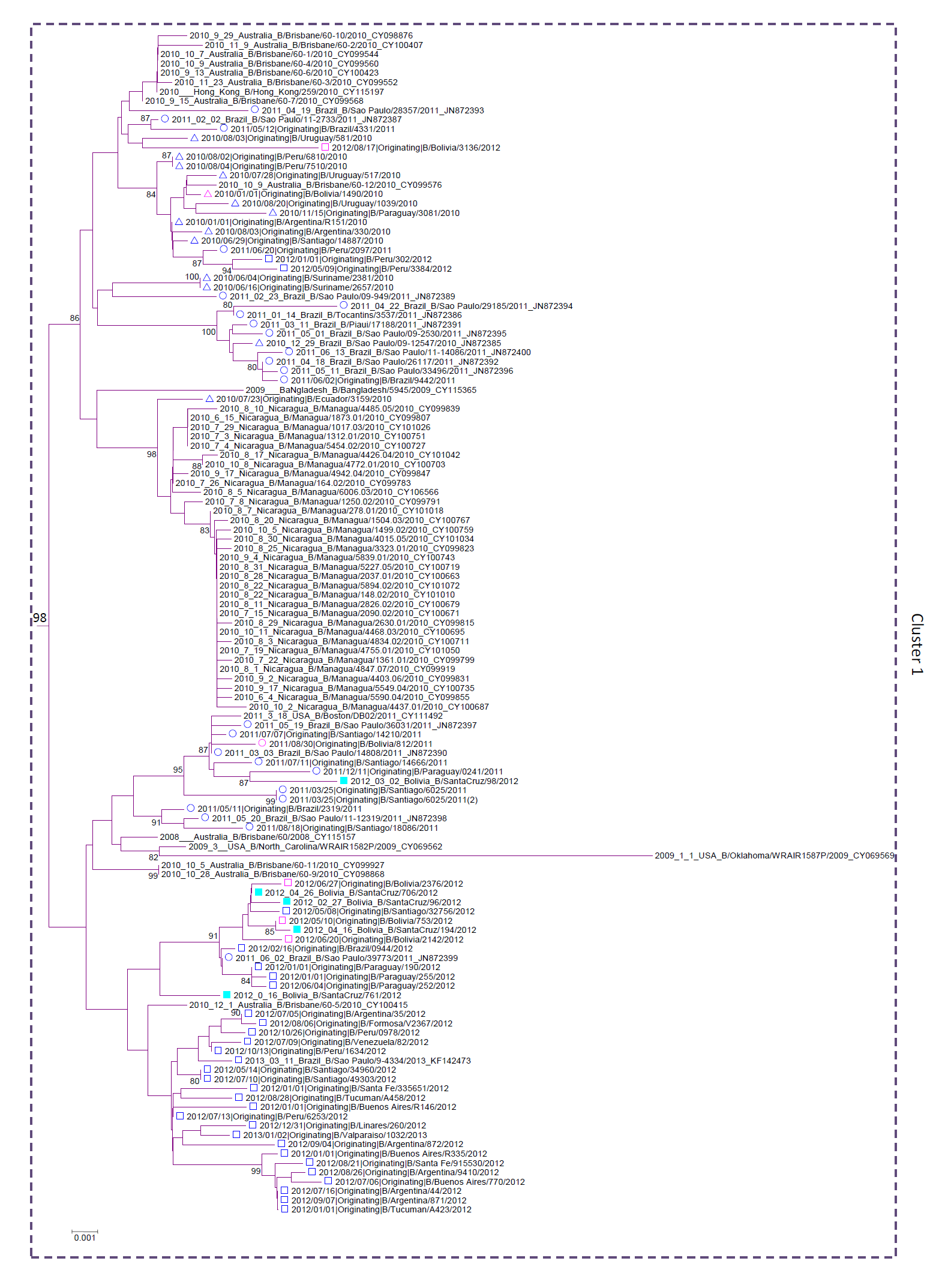

Supplement: Additional file 2 — Influenza B phylogeny cluster 1. This is a magnification of Influenza B phylogeny cluster 1 (Figure 3). Legend shape represents the year of strain isolation: square for 2012; circle for 2011; triangle with the tip up for 2010, triangle with the tip down for 2009; rhombus for <2009. The colour represents the geographical origin: light blue for Bolivian strains from this study; pink for Bolivian strains from databases; dark blue for South American strains from databases. [file 1743-422X-11-35-S2.tiff]

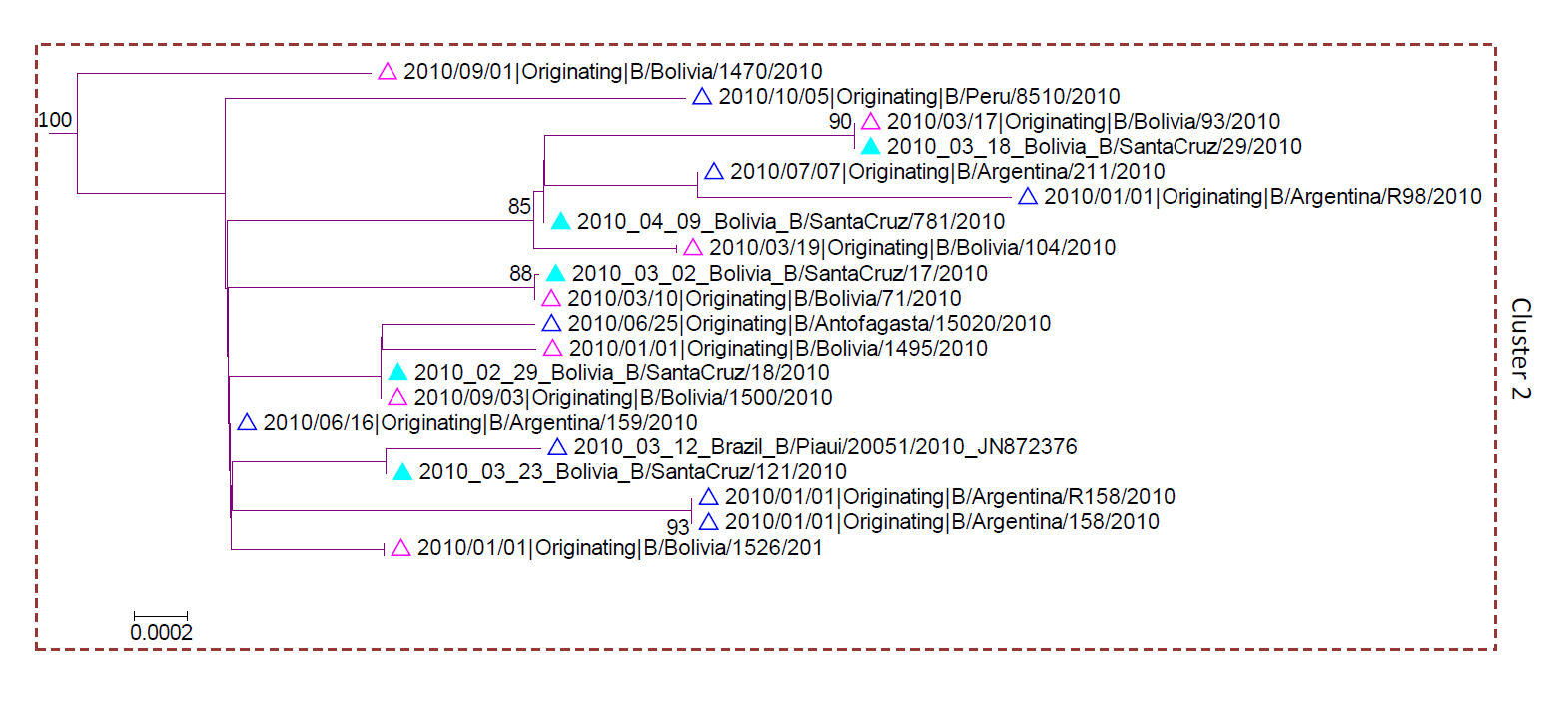

Supplement: Additional file 3 — Influenza B phylogeny cluster 2. This is a magnification of Influenza B phylogeny cluster 2 (Figure 3). Legend shape represents the year of strain isolation: square for 2012; circle for 2011; triangle with the tip up for 2010, triangle with the tip down for 2009; rhombus for <2009. The colour represents the geographical origin: light blue for Bolivian strains from this study; pink for Bolivian strains from databases; dark blue for South American strains from databases. [file 1743-422X-11-35-S3.tiff]

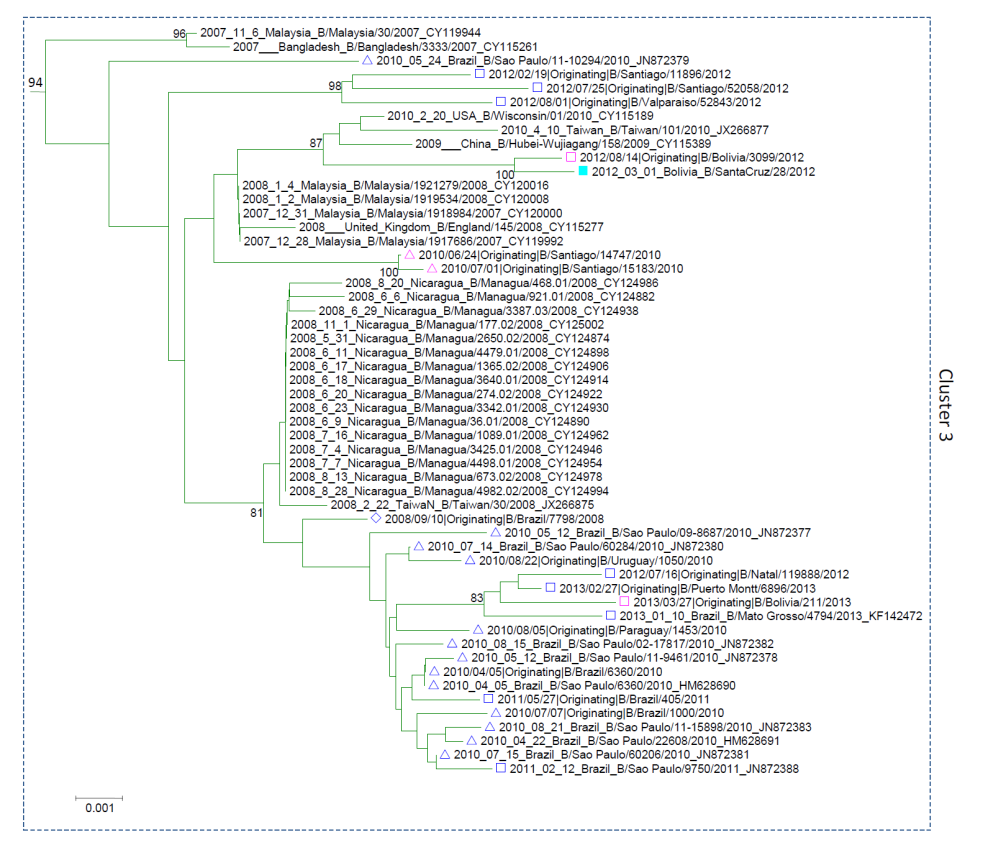

Supplement: Additional file 4 — Influenza B phylogeny cluster 3. This is a magnification of Influenza B phylogeny cluster 3 (Figure 3). Legend shape represents the year of strain isolation: square for 2012; circle for 2011; triangle with the tip up for 2010, triangle with the tip down for 2009; rhombus for <2009. The colour represents the geographical origin: light blue for Bolivian strains from this study; pink for Bolivian strains from databases; dark blue for South American strains from databases. [file 1743-422X-11-35-S4.tiff]

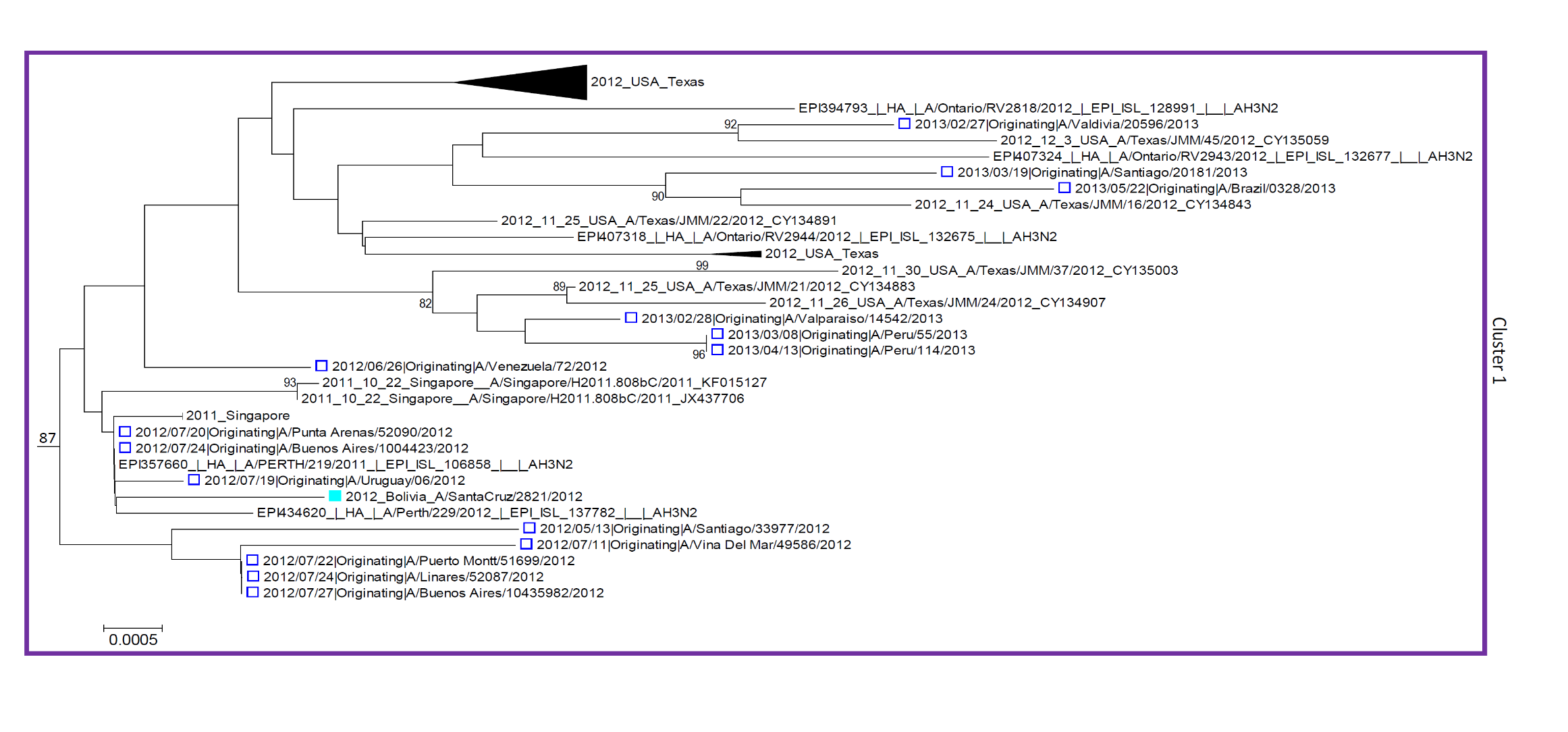

Supplement: Additional file 5 — Influenza A/H3N2 cluster 1. This is a magnification of Influenza A/H3N2 phylogeny cluster 1 (Figure 4). Legend shape represents the year of strain isolation: square for 2012; circle for 2011; triangle with the tip up for 2010, triangle with the tip down for 2009; rhombus for <2009. The colour represents the geographical origin: light blue for Bolivian strains from this study; pink for Bolivian strains from databases; dark blue for South American strains from databases. [file 1743-422X-11-35-S5.tiff]

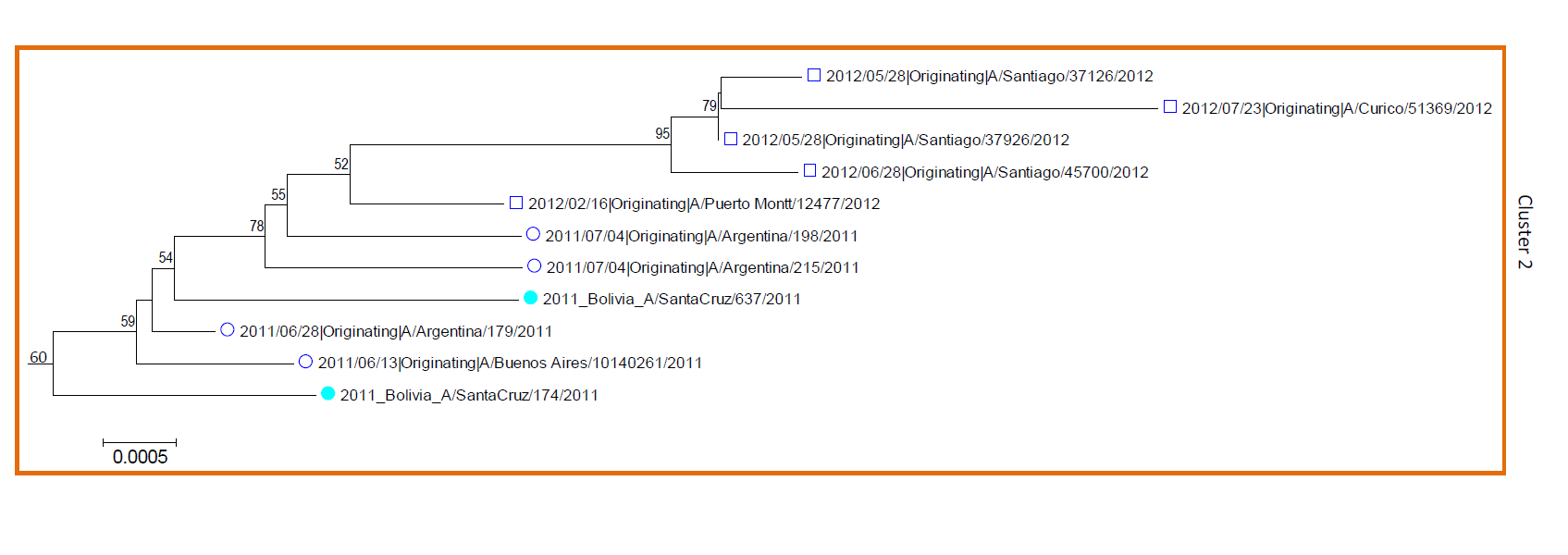

Supplement: Additional file 6 — Influenza A/H3N2 cluster 2. This is a magnification of Influenza A/H3N2 phylogeny cluster 2 (Figure 4). Legend shape represents the year of strain isolation: square for 2012; circle for 2011; triangle with the tip up for 2010, triangle with the tip down for 2009; rhombus for <2009. The colour represents the geographical origin: light blue for Bolivian strains from this study; pink for Bolivian strains from databases; dark blue for South American strains from databases. [file 1743-422X-11-35-S6.tiff]

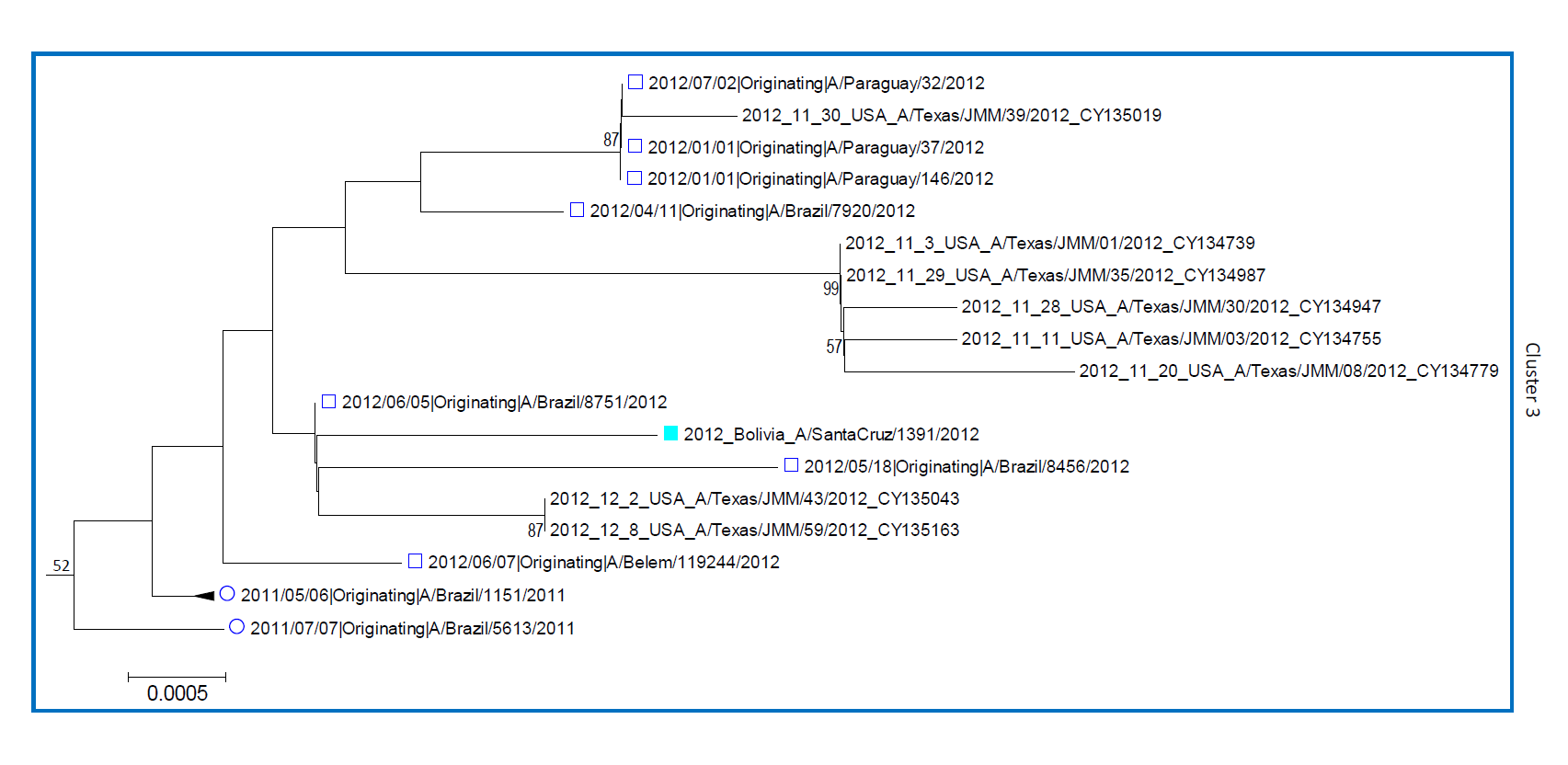

Supplement: Additional file 7 — Influenza A/H3N2 cluster 3. This is a magnification of Influenza A/H3N2 phylogeny cluster 3 (Figure 4). Legend shape represents the year of strain isolation: square for 2012; circle for 2011; triangle with the tip up for 2010, triangle with the tip down for 2009; rhombus for <2009. The colour represents the geographical origin: light blue for Bolivian strains from this study; pink for Bolivian strains from databases; dark blue for South American strains from databases. [file 1743-422X-11-35-S7.tiff]

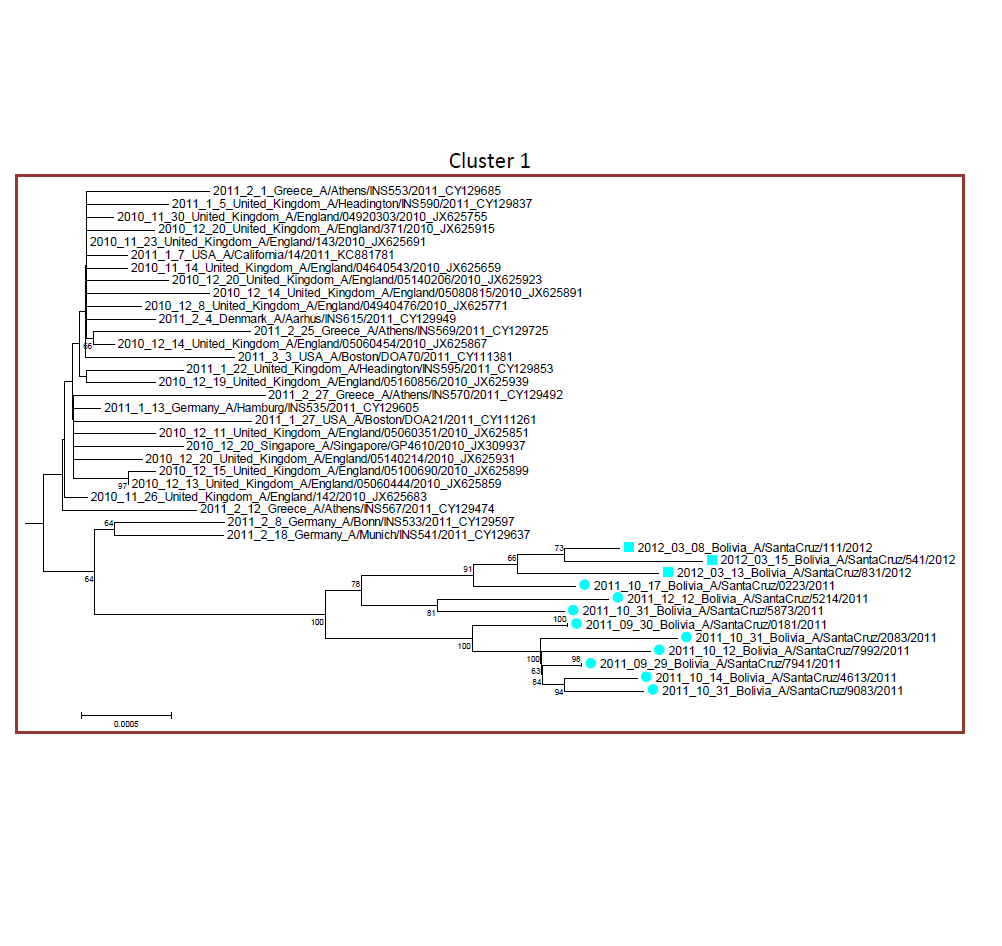

Supplement: Additional file 8 — Influenza A(H1N1)pdm09 cluster 1. This is a magnification of Influenza A(H1N1)pdm09 phylogeny cluster 1 (Figure 5). Legend shape represents the year of strain isolation: square for 2012; circle for 2011; triangle with the tip up for 2010, triangle with the tip down for 2009; rhombus for <2009. The colour represents the geographical origin: light blue for Bolivian strains from this study; pink for Bolivian strains from databases; dark blue for South American strains from databases. [file 1743-422X-11-35-S8.tiff]

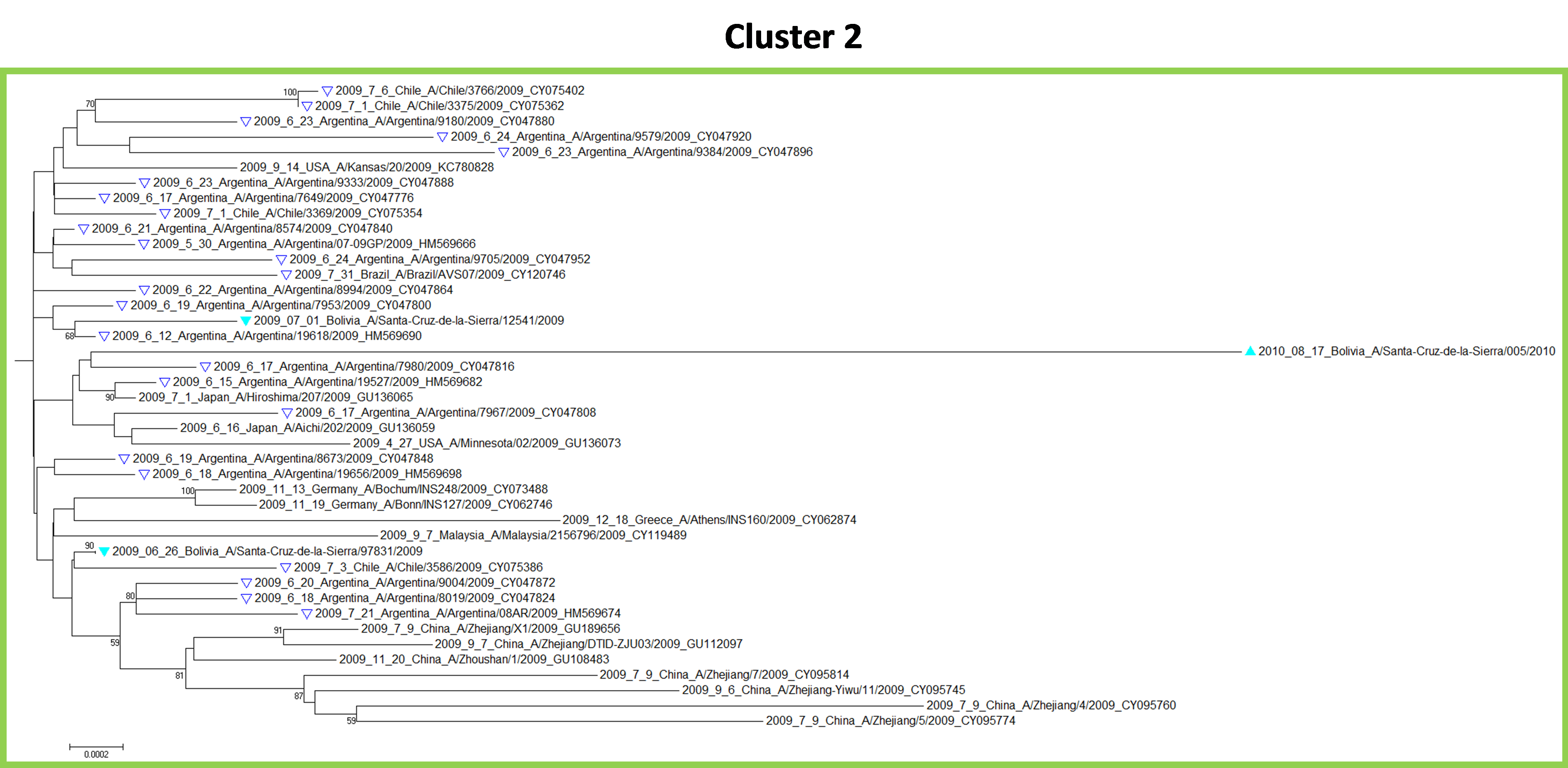

Supplement: Additional file 9 — Influenza A/H1N1 cluster 2. This is a magnification of Influenza A(H1N1)pdm09 phylogeny cluster 2 (Figure 5). Legend shape represents the year of strain isolation: square for 2012; circle for 2011; triangle with the tip up for 2010, triangle with the tip down for 2009; rhombus for <2009. The colour represents the geographical origin: light blue for Bolivian strains from this study; pink for Bolivian strains from databases; dark blue for South American strains from databases. [file 1743-422X-11-35-S9.png]

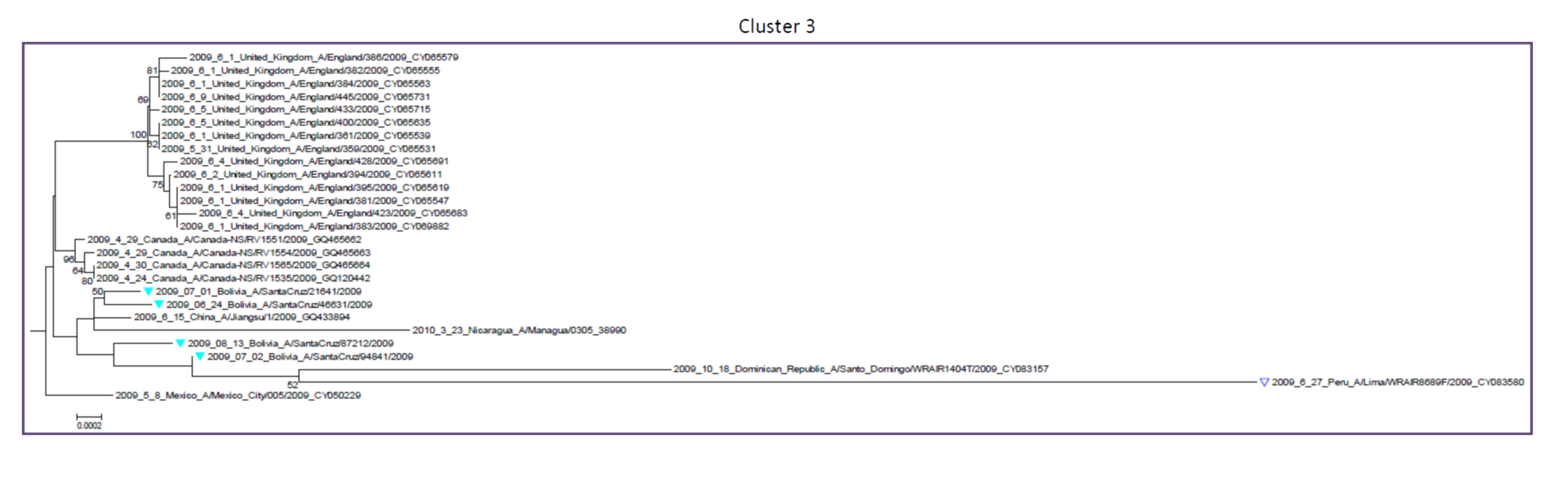

Supplement: Additional file 10 — Influenza A/H1N1 phylogeny cluster 3. This is a magnification of Influenza A(H1N1)pdm09 phylogeny cluster 3 (Figure 5). Legend shape represents the year of strain isolation: square for 2012; circle for 2011; triangle with the tip up for 2010, triangle with the tip down for 2009; rhombus for <2009. The colour represents the geographical origin: light blue for Bolivian strains from this study; pink for Bolivian strains from databases; dark blue for South American strains from databases. [file 1743-422X-11-35-S10.tiff]

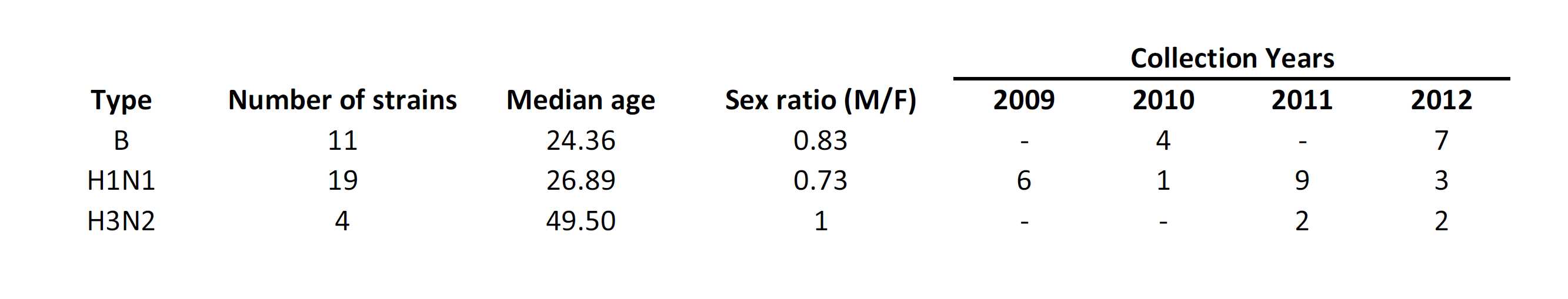

Supplement: Additional file 11 — Sequenced sample information. [file 1743-422X-11-35-S11.png]
